# Mining the diseasome

**DOI:** 10.1186/1756-0381-4-25

**Published:** 2011-09-09

**Authors:** Davnah Urbach, Jason H Moore

**Affiliations:** 1 Dartmouth College, Institute for Quantitative Biomedical Sciences, One Medical Center Dr., Lebanon, NH 03756, USA; 2 Dartmouth Medical School, Department of Genetics, One Medical Center Dr., Lebanon, NH 03756, USA; 3 Dartmouth Medical School, Department of Community and Family Medicine, One Medical Center Dr., Lebanon, NH 03756, USA

## 

Over the last ten years, genome-wide association studies (GWAS) have reported over 4000 single nucleotide polymorphisms associated to more than 200 traits [1,2]. Despite providing us with a slightly better understanding of the genetic architecture of common diseases, generating avalanches of new hypotheses, and fostering timid progress in pharmacogenomics [3], genetic associations studies haven't yet revolutionized clinical practice [4]. Hence, although such studies are still published at a remarkable pace, the notion of "post-GWAS" functional characterization of risk loci [2] is gradually gaining in popularity. Indeed, deciphering the function of disease-associated genetic variants is likely to get us closer to achieving an understanding of disease architecture that will ultimately be translatable into clinical applications. Despite this gradual change in research priorities, the field of medical genomics remains fairly conservative: the "single gene single disease" paradigm largely prevails, to the detriment of the avant-garde notion of "diseasome" [5] and of human disease network ("HDN") in particular, and attempts to truly integrate clinical information (e.g., age at onset or reduction in life span) and molecular data are scarce. Here we call for a revival of the notion of disease network, and recall how superimposing layers of clinical data and biological information to such networks may help identify novel disease genes. An inspiring read in that context is the recent paper by Barabási and coworkers on network medicine [6].

Diseases are traditionally considered as discrete entities and classified accordingly. However, the networks of genes accountable for particular disease phenotypes most certainly overlap, with individual genes simultaneously serving the cause of multiple disorders [5,7]. Clinically distinct diseases have genes in common, like nodes in a network have links in common, and DNs capture this analogy by representing diseases with nodes and the genes they share with links. In such a network representation, breast cancer and pancreatic cancer for instance are two nodes connected by TP53 [5]. What the concept of DN implies is that many susceptibility loci hitherto associated to distinct diseases are in fact likely to contribute to the genetic architecture of several disorders. Hence, rather than initiating genetic association studies with no a priori hypothesis about where in the genome to look for potential candidate risk loci, the information captured by HDNs may serve the purpose of anchoring the search for susceptibility loci in genomic regions known to harbor genetic variants predictive of other "linked" diseases. Subsequently, the human interactome [6], i.e., the compendium of molecular, phenotypic and genetic interactions, or genome-wide regulatory networks [8] can serve as maps to navigate the genome in search of further susceptibility loci.

Additional indices on where to start exploring the genome for susceptibility loci can be inferred from general principles of human diseases and clinical data. For example, a considerable fraction of diseases with onset early in life appear to result from defects in enzyme-encoding genes, whereas diseases with onset during adulthood appear to be caused by alterations in genes encoding modifiers of protein functions [9]. Thus, clinical information such as age at onset or severity can serve as valuable expert knowledge to narrow down the genomic search space to genes or genetic domains that are biologically and clinically meaningful. Additionally, and although this is not always the case, co-morbid disorders often share genes [6]. Hence, using well-established susceptibility loci for co-morbid disorders as a starting point in genetic association studies may further enhance the success rate of these endeavors.

Recent years have come with major advancements in candidate gene prioritization and our understanding of the genetic architecture of human diseases is undoubtedly progressing. Here we have suggested that biological and clinical information may serve as valuable expert knowledge for genetic association studies and that disease networks may provide useful guidance prior to and during data mining.
